# Trans-national conservation and infrastructure development in the Heart of Borneo

**DOI:** 10.1371/journal.pone.0221947

**Published:** 2019-09-18

**Authors:** Sean Sloan, Mason J. Campbell, Mohammed Alamgir, Alex M. Lechner, Jayden Engert, William F. Laurance

**Affiliations:** 1 Centre for Tropical Environmental and Sustainability Science, College of Science and Engineering, James Cook University, Cairns, Queensland, Australia; 2 School of Environmental and Geographical Sciences, University of Nottingham Malaysia Campus, Jalan Broga, Semenyih, Selangor, Malaysia; 3 Mindset Interdisciplinary Centre for Environmental Studies, University of Nottingham Malaysia Campus, Jalan Broga, Semenyih, Selangor, Malaysia; Universiti Malaysia Sabah, MALAYSIA

## Abstract

The Heart of Borneo initiative has promoted the integration of protected areas and sustainably-managed forests across Malaysia, Indonesia, and Brunei. Recently, however, member states of the Heart of Borneo have begun pursuing ambitious unilateral infrastructure-development schemes to accelerate economic growth, jeopardizing the underlying goal of trans-boundary integrated conservation. Focusing on Sabah, Malaysia, we highlight conflicts between its Pan-Borneo Highway scheme and the regional integration of protected areas, unprotected intact forests, and conservation-priority forests. Road developments in southern Sabah in particular would drastically reduce protected-area integration across the northern Heart of Borneo region. Such developments would separate two major clusters of protected areas that account for one-quarter of all protected areas within the Heart of Borneo complex. Sabah has proposed forest corridors and highway underpasses as means of retaining ecological connectivity in this context. Connectivity modelling identified numerous overlooked areas for connectivity rehabilitation among intact forest patches following planned road development. While such ‘linear-conservation planning’ might theoretically retain up to 85% of intact-forest connectivity and integrate half of the conservation-priority forests across Sabah, in reality it is very unlikely to achieve meaningful ecological integration. Moreover, such measure would be exceedingly costly if properly implemented–apparently beyond the operating budget of relevant Malaysian authorities. Unless critical road segments are cancelled, planned infrastructure will fragment important conservation landscapes with little recourse for mitigation. This likelihood reinforces earlier calls for the legal recognition of the Heart of Borneo region for conservation planning as well as for enhanced tri-lateral coordination of both conservation and development.

## 1.0 Introduction

Road infrastructure expansion across the Global South is increasingly recognized as a key factor in regional conservation and development planning [1–3], on par with demographic growth, urbanization, and climate change. Globally, road length is projected to increase ~20–60% by 2050, with the vast majority anticipated in the Global South [4, 5]. Such increases reflect high demographic and economic growth, the devolution of governance to local levels where road building is favored [6, 7], and the advance of infrastructure mega-projects to open underdeveloped regions to global markets [8–10]. Infrastructure mega-projects in particular are expected to encroach upon intact ‘wilderness’ areas in many regions [4], as typified by the Chinese Belt and Road Initiative [10, 11] and economic-corridor schemes in eastern Indonesia [12].

Globally, the proliferation of infrastructure mega-projects in the Global South coincides with an increasing prioritization of regional-scale integrated conservation. Internationally, parties to the Convention on Biological Diversity (CBD) have committed to 17% national coverage of “ecologically representative and well-connected systems of protected areas” by 2020 [13], an increase of 7% relative to an earlier CBD target. Across the Global South, Juffe-Bignoli *et al*. [14] identify shortfalls of 3–5% relative to the current target for all regions but Latin America, while Saura *et al*. [15] indicate consistently greater shortfalls of 2–15%, particularly in Asia. However, in Sabah, Malaysia, the focus of this study, an ambitious embrace of the CBD via the aligned Sabah Biodiversity Strategy [16] is fulfilling various CBD targets. Prominent goals within this Strategy include expanded protected-area coverage to >20%, the protection of key habitats outside of protected areas via enhanced forest connectivity, and the conservation of biodiversity-rich landscapes via cooperation with neighboring countries and states [16]. Indeed, for both Sabah and the Global South generally, international cooperation is increasingly necessary to realize enhanced regionally-integrated conservation [15, 17–19]. Correspondingly, the number of trans-boundary conservation areas has more than tripled internationally during the last three decades [20]. Nonetheless, achieving enhanced protected-area coverage while neglecting losses to regional connectivity posed by infrastructure mega-projects will prove insufficient to maintain biodiversity and ecosystem resilience [21].

**Fig 1:**
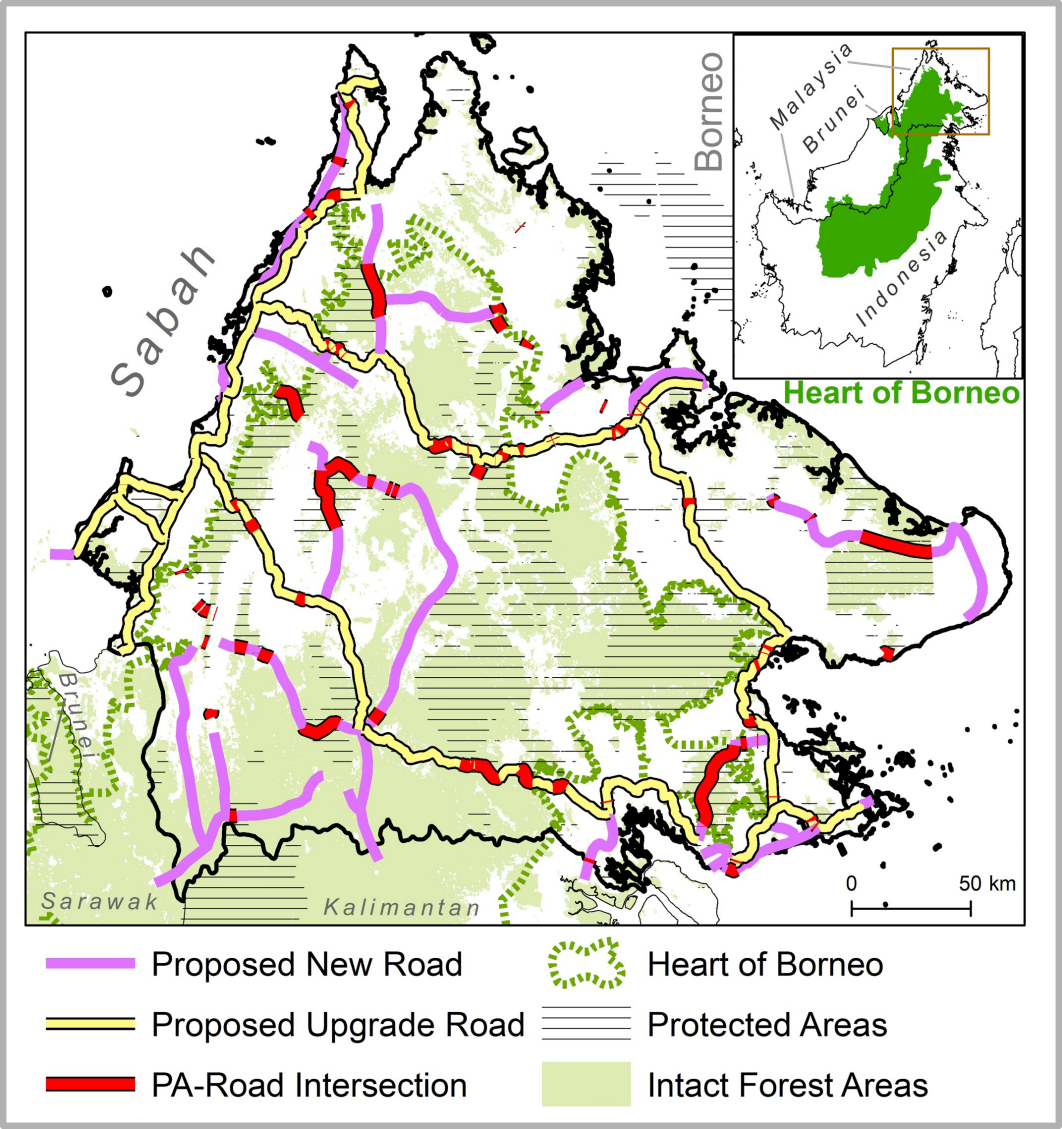
The Heart of Borneo trilateral conservation region (inset) and extent of proposed new and upgrade roadways in Sabah according to the Sabah Structure Plan 2033, displayed relative to intact forests. Notes: Intact Forest Areas and Protected Areas are as defined by this study. See S1 Fig for additional information on official forest-use designations.

The Heart of Borneo initiative (HoB) is indicative of the potential of regionally-integrated conservation schemes to enhance national protected-area coverage. Established in 2007, the HoB formalized cooperation between Malaysian Borneo (Sabah and Sarawak states), Indonesian Borneo (four provinces of Kalimantan), and Brunei to integrate and enhance a 22-million hectare (Mha) trans-boundary network of protected areas (PAs), production forests, and other sustainable forest uses for mutual conservation benefit (Fig 1). The HoB has been particularly fruitful in Sabah, where it covers 54% of the state’s territory and is acknowledged within the Sabah Biodiversity Strategy [16]. There, protected areas (PAs) have since expanded from 12% to 26% of the state’s extent, in keeping with the CBD targets [22]; >0.15 Mha of forests have been restored [23]; reduced impact logging has been adopted in all logging reserves (cf. [24]) (S1 Fig); and PA connectivity has increased both locally and regionally [25]. Hence, for Malaysia as a whole, inter-connected PAs account for 8–12% of its territorial extent (accounting for inherent territorial discontinuities [26]) and a relatively large proportion of this connectivity is dependent on trans-boundary PAs and nearby PAs in adjacent countries [15].

Regional, trans-boundary conservation initiatives such as the HoB are however arguably especially vulnerable to large-scale infrastructure development schemes. On the one hand, the HoB initiative is inherently contingent on international collaboration and territorial integration, as where the full benefit of PAs in a given administrative area depends on the sound management of forests in an adjacent area. Trilateral cooperation in both conservation and development is therefore key. On the other hand, large-scale infrastructure schemes are inherently unilateral, both with respect to the sovereignty of their design and implementation but also their political-administrative insulation from state-level forest management. Thus, in Sabah, Sarawak, and Kalimantan, large-scale development schemes driven by federal economic agenda struggle to reconcile with the HoB. The Sabah Development Corridor, driven by successive Malaysian Plans for industrial development, seeks to quadruple Sabah’s GDP over 2008–2025, partly by expanding a Pan-Borneo highway network throughout the HoB (Fig 1). Similarly, planned road developments in Kalimantan, driven by the Acceleration and Expansion of Indonesia masterplan [27], would transect 1920 km of the Heart of Borneo ‘spine’ along the Malaysian border [28]. In this context, the incautious pursuit of large-scale development schemes by even a single state may have disproportionate negative effects for regionally-integrated conservation.

Here we describe anticipated changes to the integrity of the HoB that would follow the planned Pan-Borneo network development in Sabah. We highlight the potential impacts of the Pan-Borneo highway on the connectivity of protected areas and intact forests within the northern HoB as well as assess mitigation opportunities. Findings call for improved trilateral conservation planning and highlight a reliance on ‘linear-conservation’ approaches to mitigate planned developments. Recommendations for improved conservation-and-development planning are discussed in the context of infrastructure mega-projects.

## 2.0 Materials and methods

### 2.1 Protected area connectivity

#### 2.1.1 Counts and areas of protected areas

For intact forest patches in the HoB (Fig 1), we observed the number and extent of protected areas (PAs) inter-connected by intact forest cover before and after planned road developments in Sabah. Planned new road and planned road upgrades encompass Sabahan segments of the Pan-Borneo Highway as well as supplementary roads according to the Sabah Structural Plan 2033 [29]–an overarching statutory plan guiding development planning at local administrative levels.

**Fig 2:**
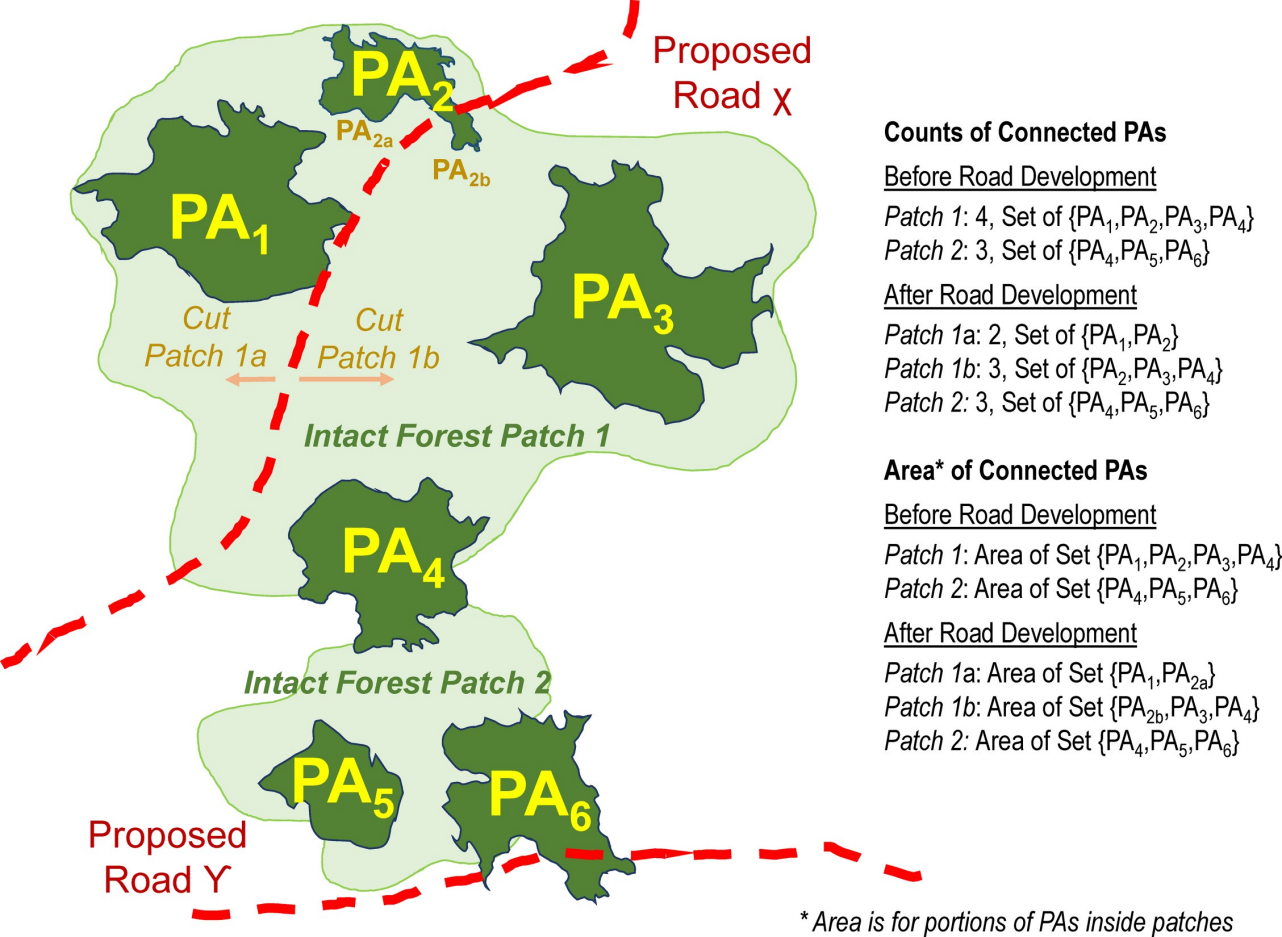
Hypothetical schematic of estimates of counts and extents of inter-connected protected areas (PAs) by intact forest patches, before and after road development. Note: PA_4_ bridges Intact Forest Patch 1 and Intact Forest Patch 2 but does not connect their respective sets of PAs due to the local absence of intact forest. Proposed Road ‘X’ bisects Intact Forest Patch 1 and PA_2_ into Patch 1a and Patch 1b as well as PA_2a_ and PA_2b_, respectively. This bisection of PA_2_ does not affect the count of PAs in the resultant Patch 1b (3 PAs) but it does affect the PA extent in Patch 1b by confining PA_2a_ within Patch 1a. The extent of PAs per patch is estimated exclusively for portions of PAs within a patch. Proposed Road ‘Ƴ’ bisecting PA_6_ would affect neither the count nor extent of inter-connected PAs of Intact Forest Patch 2 because the road is outside the patch.

PAs wholly within or partially within an intact forest patch were considered inter-connected across that patch (Fig 2). The number and extent of inter-connected PAs per current intact-forest patch were observed according to the overlap of spatial data delineating PAs and intact-forest patches using ArcGIS, as discussed below. Rarely, a single PA partially overlapped more than one intact forest patch and thus was a member of more than one set of inter-connected PAs (Fig 2: PA_4_). Such a PA alone could not connect such sets of PAs to each other in the absence of intermediary intact forest, however.

To model changes to PA connectivity following road development, planned new and upgrade roadways bisecting an intact forest patch constituted interruptions to the connectivity amongst PAs within that patch on either site of the bisecting roadway (Fig 2: PA_1_ disconnected from PA_3_ PA_4_). Planned new and upgrade roadways were buffered by 1 km and areas of current intact forest patches coincident with the buffer were removed from the extent of intact forest. Thus, an intact forest patch bisected by a buffered roadway would become two intact forest patches. The number and extent of inter-connected PAs amongst these subdivided, post-development intact forest patches were calculated in the same way as for the current intact forest patches. The 1 km buffer distance accounts for local ecological effects and human activities along roadways [30–35].

PAs were treated as indivisible when determining counts of inter-connected PAs per intact forest patch in order to ensure unambiguous counts before and after road development. Therefore, where a road through a PA would bisect its intact forest patch, the PA would remain integral and count towards the set of PAs in each resultant forest patch (Fig 2: PA_2_ counts towards Patch 1a and new Patch 1b). To account for the bisection of PAs, we estimated changes to total inter-connected PA extent per intact forest patch before and after road development. These areal estimates consider only portions of PAs within an intact forest patch and treat PAs as divisible by roadways (Fig 2: PA_2a_ but not PA_2b_ is in Patch 1a; the middle and bottom portions of PA_4_ and PA_6_ are respectively excluded).

#### 2.1.2 Planned road infrastructure development

We digitized 1295 km and 1337 km of planned new roads and planned road upgrades respectively (Fig 1) from the Sabah Structural Plan 2033 [29]. A 1:500,000 map of planned roadways [36] was digitized and georeferenced to Sabahan administrative boundaries according to GADM v2.1 spatial data [37]. Planned roadways were then manually digitized on-screen in ArcGIS at viewing scales of 1:5000 to 1:10,000. The digitized routes for planned upgrade roadways were subsequently overlaid on high-resolution imagery in Google Earth, in which the routes of existing roadways nominated for upgrade were visible. Comparisons of these existing routes to the digitized planned upgrade roadway routes found them to be separated by < 300 meters generally. This is an appropriate distance and indicative of accurate digitization, considering that road upgrades entail the construction of four-lane highways parallel to existing dual-lane roads, in addition to the conversion of rudimentary dirt tracks to paved highways. The fragmentation of the HoB due to road developments reported here is best understood as an acute exacerbation of ongoing fragmentation, considering the nature of road upgrades.

#### 2.1.3 Protected areas

PAs were defined by the World Database on Protected Areas [38] and supplemented by additional ‘totally protected’ forest designations of the Sabah Forestry Department (e.g., protection forest reserves, virgin jungle reserves, wildlife reserves and conservation sanctuaries) (S1 Fig). Analyses considered PAs wholly or partially within the HoB boundaries. Following Sloan *et al*. [9] and Laurance *et al*. [8], overlapping PAs were ‘dissolved’ into discrete polygons to avoid double-counting overlapping PAs of distinct designations for a common area (e.g., national park, World Heritage Site).

#### 2.1.4 Intact forest patches

Intact forest patches were defined as areas of either contiguous lowland forest, lower montane forest, montane evergreen forests, or peat swamp forest as classified by Miettinen *et al*. [39] using MODIS imagery for 2015 with 250-m pixel resolution. Validations of these forest classes via visual interpretations of satellite imagery, as described by Miettinen *et al*. [39], confirm them to be “predominantly primary (including degraded) forests of >60% canopy cover and to occasionally include mature secondary forests that have attained structural characteristics similar to primary forests”. Validation entailed visual interpretations of 1000 250-m MODIS pixels across 10 Landsat-8 false-color images spanning tropical Southeast Asia. Additionally, 400 pixels were interpreted using high-resolution (~1 m) true-color imagery across the extents of the mixed forest regrowth/plantation and industrial-plantation classes. User accuracies for the forest classes comprising our intact forest areas were 90–91%.

Our delineation of intact forests patches is possibly conservative, given its exclusion of the mixed regrowth/plantation, mosaic, and industrial-plantation classes observed by Miettinen *et al*. [39]. While these mixed tree-cover classes might permit limited connectivity between intact forest patches, in Sabah these classes are generally highly intervened and associated with agriculture, including oil palm, as confirmed by the validations performed by Miettinen *et al*. [39] and our independent visual inspections of the mixed classes using high-resolution imagery in Google Earth. Intact forest patches are more liberal in terms of their contiguity, as narrow, sub-pixel gaps in the forest canopy due to existing small roadways may be unobserved in the MODIS classification. Any such unobserved gaps in an intact forest patch were presumed to be inconsequential for connectivity across that patch.

### 2.2 Intact forests structural connectivity

We assessed changes to structural connectivity across intact forest patches, presuming a range of potential faunal movements between patches and scenarios for crossing planned new and upgrade roadways. Spatial networks of intact forest patches and inter-patch linkages representing hypothetical faunal dispersal routes [40, 41] were defined for a post-development landscape in which intact forest patches were already bisected by planned new and upgrade roads (buffered by 1 km). The post-development landscape encompasses Sabah plus a surrounding 20-km buffer zone, extending into northern Kalimantan and eastern Sarawak, allowing for the consideration of trans-boundary forest connectivity.

We assessed connectivity using a graph-theoretic mathematical approach [42, 43] treating the landscape as a network of intact forest patches of ≥ 10 ha and linkages between such patches. Routes of potential faunal movement were defined between patches across the post-development landscape. Drawing on these routes, discrete patch-linkage networks were ultimately defined according to pre-specified maximum inter-patch faunal dispersal distance thresholds. A single patch-linkage network is comprised of patches linked to each other yet isolated from the patches of other patch-linkage networks. A range of inter-patch faunal dispersal distance thresholds were considered during network modelling, from nil to a maximum distance yielding complete connectivity amongst all intact-forest patches across the post-development landscape (S2 Fig). For each dispersal distance threshold, we recorded the corresponding number of patch-linkage networks across the post-development landscape as well as the integral index of connectivity (IIC) (S2 Fig). The IIC describes the average ‘accessibility’ of intact forest patches across the post-development landscape. Conceptually, the IIC describes the probability that two fauna randomly located within intact forest patches may exchange places with each other while respecting the pre-specified dispersal distance threshold. Full mathematical details on the IIC are provided elsewhere [44, 45]. We excluded patches < 10 ha to facilitate the computationally-intensive modelling. Excluded patches accounted for 6.3% of all intact forest patches in the post-development landscape but only 0.02% of their extent.

We explored differences in the regional distributions of key inter-patch linkages amongst two scenarios of road development varying according to the permeability of planned roadways to faunal movements. Both scenarios adopt a 2-km dispersal distance threshold to define linkages between intact forest patches. Only one scenario allows inter-patch linkages to span planned roadways where patches are < 2 km of each other. Linkages across planned roadways are precluded in the other scenario. The 2-km dispersal distance threshold was adopted because it (*i*) marginally exceeded the localized road effects [35], (*ii*) constituted a lower ‘stable’ threshold above which IIC values and the number of patch-linkage networks changed relatively gradually (S2 Fig), and (*iii*) was considered generally applicable to most vertebrates in the absence of local species-specific data. Differences in the distributions of key linkages between these two scenarios highlight the degree to which planned roadways would disconnect major intact forest areas that are adjacent to planned roads and/or bisected by such roads. For each scenario, the importance of each individual linkage for overall regional connectivity was estimated by iteratively removing the linkage and re-calculating the IIC via the Graphab Delta-Metric tool [44, 45]. Larger resultant deviations to the IIC, termed ΔIIC, indicate that a given linkage is more important for connectivity across the post-development landscape. Key linkages are those 15 linkages with the greatest ΔIIC values. These linkages comprise ~0.5% of all linkages but 50–90% of the sum of all ΔIIC values across the post-development landscape, depending on the scenario.

The distribution of these key linkages were considered relative to priority conservation forests observed by the Sabah Structure Plan 2033 [29, 46–48]. These priority conservation forests are ‘optimal’ modelled conservation priority areas in that they satisfy pre-specified conservation targets at minimal overall societal costs. The conservation targets entail coverage of (*i*) >30% historic areas of all forest eco-types (in keeping with the Sabah’s commitment to maintain 30% of its extent under protected forest [29, 48]), rising to >75% for mangroves; (*ii*) >60% of historic ranges for select endangered mammals (banteng, clouded leopard, elephant, gibbon, orangutan, proboscis monkey, and sun bear) as well as select endemic tree species, rising to 100% for select critically endangered endemic tree species [48]. Societal costs describe the difficulty, conflict, and efficiency entailed by conservation at a given location, and are represented by (*i*) the human-footprint index [49], describing the intensity of settlement and land use; (*ii*) species richness, describing the number of species present; and (*iii*) forest-carbon stock [50], representing the impact of forest conversion on atmospheric carbon emissions. Values for species richness and carbon stock were inverted to reduce the ‘cost’ of including high-richness, high-carbon areas within the priority conservation forests. Severely degraded forests [51] were excluded from consideration. Priority conservation-forest areas were delineated by WWF-Malaysia using the Marxan conservation-optimization software [52], as described by Tai *et al*. [48], Abram *et al*. [46] and Sloan *et al*. [53], and were provided by WWF-Malaysia.

### 2.3 Opportunities for conservation mitigation

Indicative sites for potential forest corridor and roadway-underpass development were identified to explore plans for enhancing the connectivity of intact forests and PAs across the northern HoB region. Indicative sites are those that would greatly enhance the structural connectivity of intact forests and PAs across the post-development landscape, being again comprised of Sabah and the 20-km buffer extending into Kalimantan and Sarawak. Each site integrates intact forest patches, typically as pairs, with at least one patch having relatively high connectivity importance (ΔIIC values for linkages permitted to span roadways), larger PA extent, and/or large intact forest extent (S3 Fig; S1 Table), in order of priority. These three criteria tended to covary and flag relatively few locales, such that the sites were readily identified ‘opportunistically’ rather than via pre-specified thresholds for the three criteria. Site selection also reflected three secondary criteria considering the feasibility and efficacy of the indicative sites, namely: (*i*) relatively short linkages (< 2km) amongst adjacent intact forest patches, as to facilitate hypothetical corridor/underpass development; (*ii*) extensive areas of contiguous forest relatively unsettled and free of agricultural development between the intact forest patches, as confirmed by visual inspections of high-resolution imagery in Google Earth; and (*iii*) regional synergy, such that a set of individual sites may link a series of important patches to each other across Sabah. In total, 12 indicative sites were identified (S1 Table).

## 3.0 Results

### 3.1 Protected area connectivity

**Fig 3:**
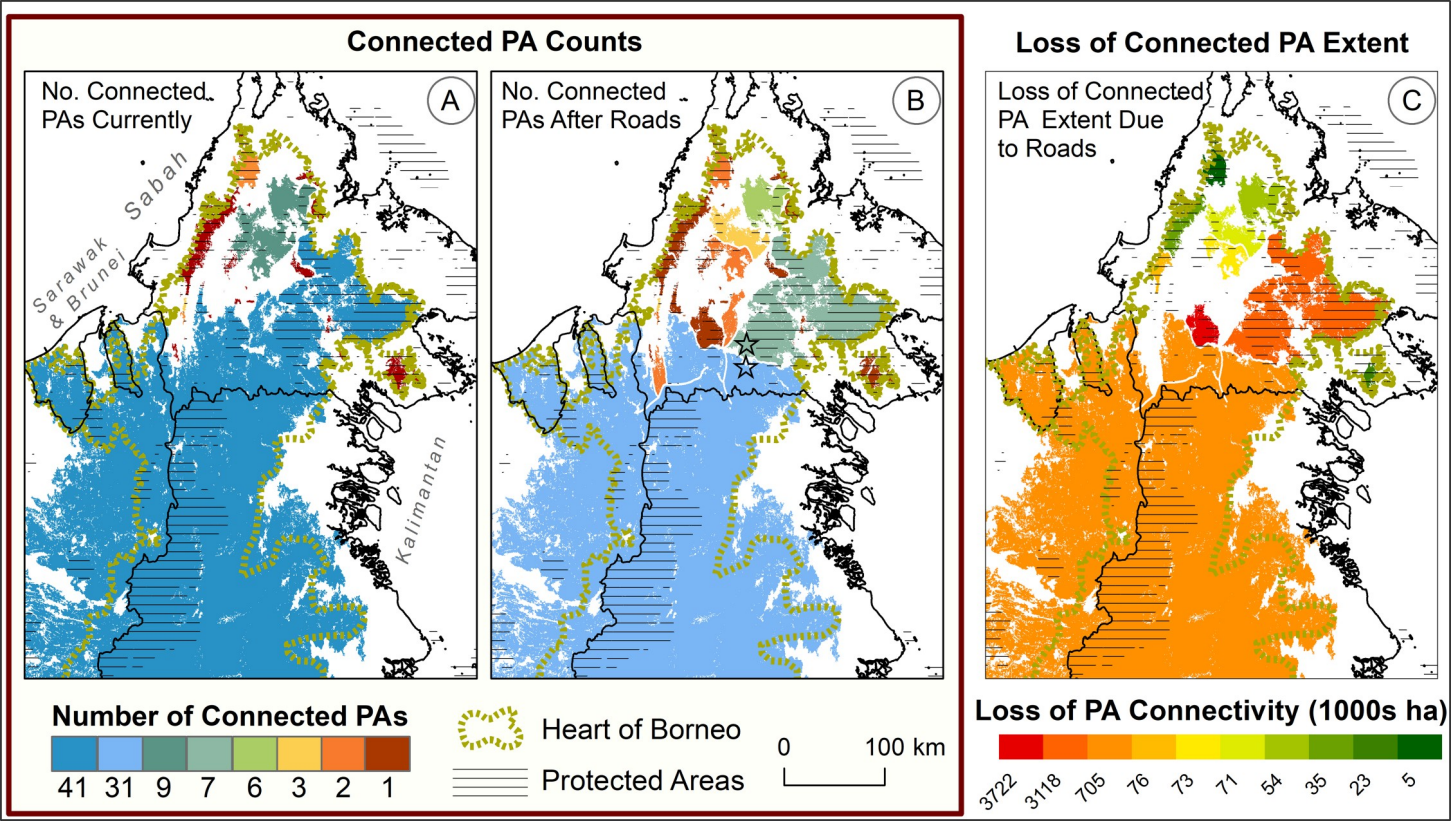
**Number of inter-connected protected areas (PAs) per intact forest patch in the northern Heart of Borneo region, (A) currently and (B) after planned road developments (center), as well as (C) related losses of inter-connected PA extent per patch due to planned road developments (right).** Note: Panels A and B show ‘before’ and ‘after’ scenarios regarding planned road development, while panel C shows anticipated changes due to the planned development relative to the present. In Panel B, two grey stars denote the two main post-development patches descendent from the current trans-boundary patch, namely the ‘confined’ trans-boundary patch in southern Sabah and the large patch in Kinabatangan and Lahad Datu districts to its north. Panel C shows changes for the 10 post-development intact forest patches having the greatest post-development PA extents. The losses of inter-connected PA extent in Panel C are therefore not necessarily the greatest absolute losses pending. Absolute losses may be greater for some patches entirely disconnected from PAs by planned road development.

Most PA connectivity in the HoB is concentrated in a single intact forest patch spanning southern and eastern Sabah, northern and central Kalimantan, eastern Sarawak, and Brunei (Fig 3A). This trans-boundary patch hosts 41 PAs, accounting for half of the PAs in the HoB and 89% of their extent (Fig 3A). Of these 41 PAs, 19 occur at least partially inside Sabah and account for half of the PA extent coincident with the HoB.

**Fig 4:**
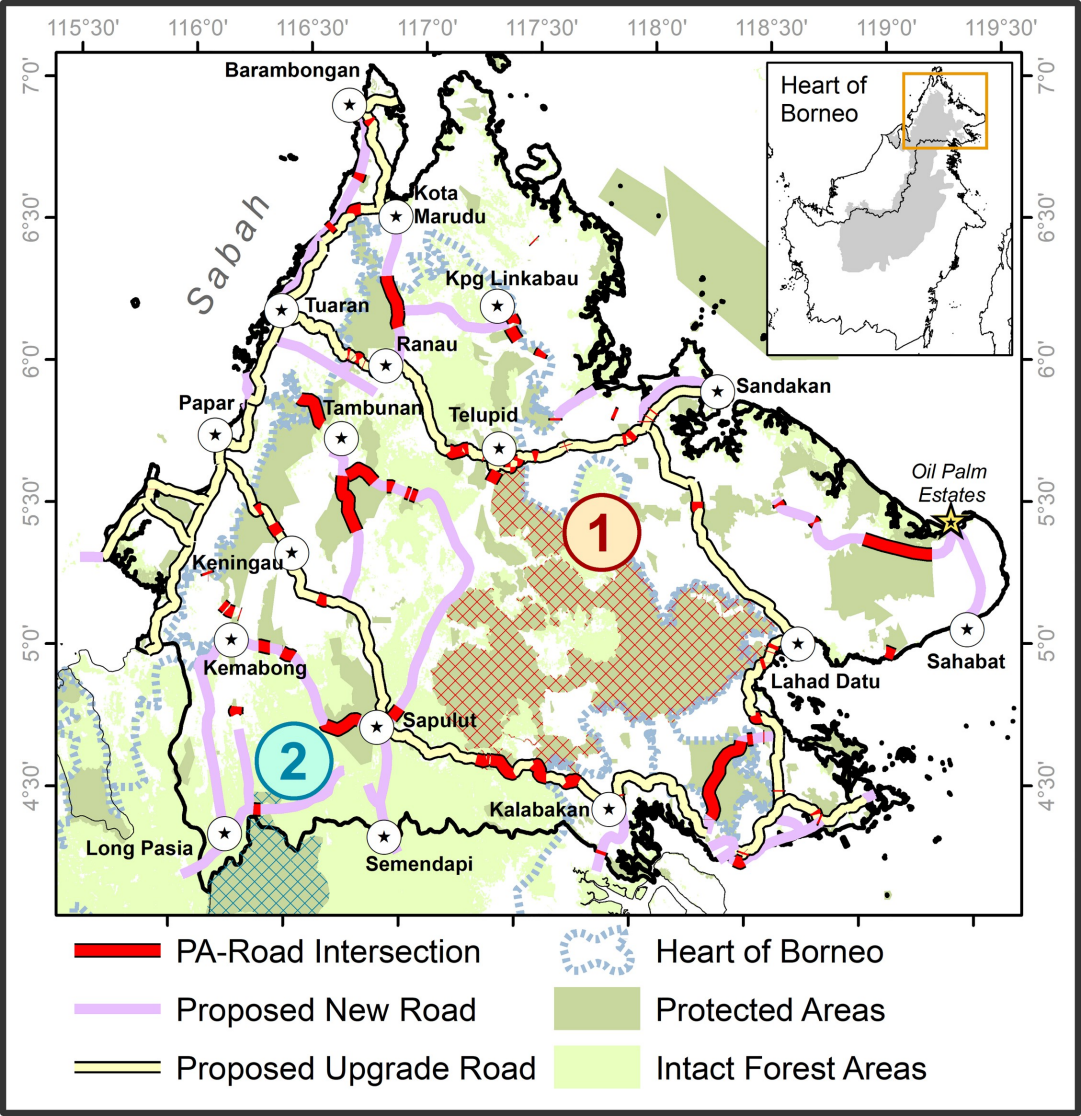
Two main agglomerations of protected areas (numbered and hatched) pending disconnection in Southern Sabah due to planned road developments amongst townships spanning the major trans-boundary patch, especially the planned Sapulut-Kalabakan route.

Planned road developments in southern Sabah would significantly fragment this trans-boundary patch and interrupt PA connectivity across the northern HoB (Fig 3A and 3B). Within the current trans-boundary patch, roughly half of its 4.2 Mha of PAs are comprised by two PA agglomerations immediately north and south of planned developments between the towns of Kemabong, Sapulut, and Kalabakan (Fig 4). Upon separating these two PA agglomerations, the losses of inter-connected PA extent would be >3 Mha for each the 14 intact forest patches that would result from planned road developments across southern Sabah (Fig 3C).

Planned road development would confine the current trans-boundary patch to far southern Sabah (Figs 3B and 4). This confined patch would inter-connect 10 fewer PAs than currently across Sabah and the larger HoB, with most of its 31 PAs being mostly outside Sabah. Consequently, the northern reach of the current trans-boundary patch would, as a newly isolated post-development patch immediately north of planned developments between Sapulut and Kalabakan (Fig 4), constitute the most significant patch entirely within Sabah (Kinabatangan and Lahad Datu districts) in terms of intact forest area (876 thousand hectares [Kha]), PA extent (642 Kha), and PA count (7) (Fig 3B and 3C).

### 3.2 Intact forest structural connectivity

**Fig 5:**
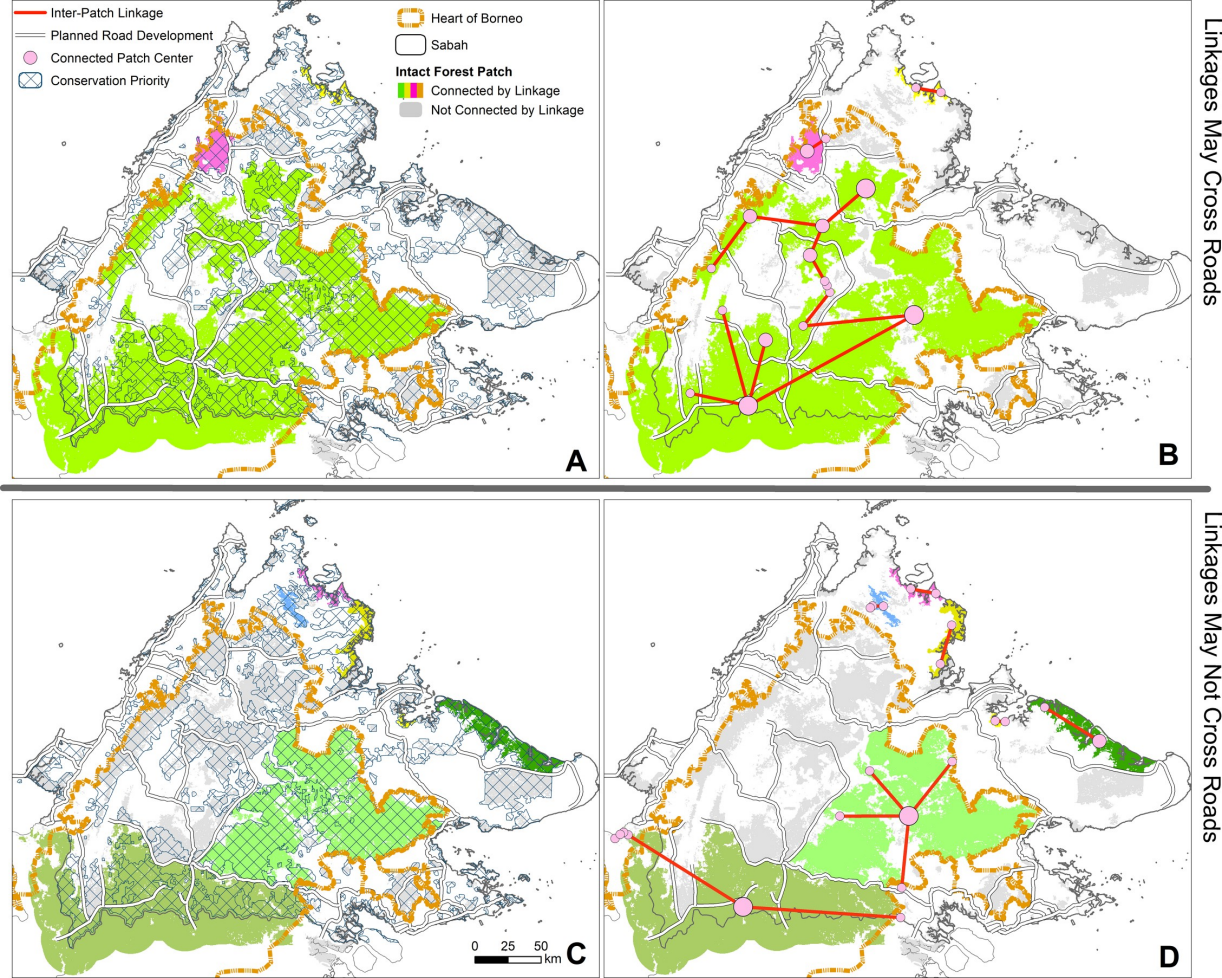
**The 15 most important linkages for structural connectivity of intact forests across Sabah, northern Kalimantan, and eastern Sarawak, alongside intact forest patches and priority conservation areas, where linkages can (A, B) or cannot (C, D) cross planned roadways to connect proximate road-adjacent forests. Intact forest patches of a given single network are displayed in shades of green, pink, yellow and blue.** Notes: The size of circular symbols for connected intact forest patches are indicative of patch area. Planned road developments are according to the Sabah Structure Plan 2033. Minor post-facto adjustments by regional conservation experts incorporated select corridors, Nipah palm areas, and remnant forest of the Lower Kinabatangan area into the priority conservation forests.

The two post-development scenarios varying by planned roadway permeability exhibit major differences in their distributions of key inter-patch linkages across the northern HoB. In the scenario in which linkages may span roadways to a limited degree (<2 km), the key linkages largely circumscribe the extent of conservation-priority forest (Fig 5A). Notably, these linkages define a single Sabah-wide network incorporating most contiguous forests in the northern HoB (78%) and thus half of the conservation-priority forests in Sabah (49%) (Fig 5B). Key linkages would cross planned roadways in all instances to define this network, with the partial exception of the linkage from the Crocker Range National Park where an existing road would be crossed instead. These key linkages thus extend to relatively large, road-adjacent intact forest areas otherwise separated by planned roadways (mean patch area: 151 Kha; per linkage: 181 Kha).

In contrast, for the scenario in which linkages cannot span planned roadways, key linkages adopt an inferior configuration in which conservation-priority forest remains disjointed. These linkages center on only two or three main post-development patches, notably including descendants of the current trans-boundary patch (Fig 5C). Further, they extend to comparatively-insignificant surrounding patches, most of which would be unaffected by planned roadways. The key linkages thus incorporate a lesser 64% and 39% of post-development intact forest cover and conservation-priority forests, respectively, with the vast majority of these areas comprised of the main patches and remaining disjointed across seven patch-linkage networks (Fig 5D). Key linkages would therefore integrate relatively little intact forest beyond the main patches (mean patch area: 102 Kha; per linkage: 150 Kha) and entirely fail to incorporate priority conservation areas across central and northern Sabah (Fig 5C).

Each scenario is a hypothetical extreme: planned roadways are neither completely permeable nor completely impermeable to faunal movements. Contrasts between the scenarios do not advocate that linkages *should* span planned roadways or where, but rather they illustrate how planned roadways are largely situated within major forest regions key to regional connectivity. Accordingly, key linkages invariably span planned roadways to maximize connectivity where allowed–something that is unlikely in practice. Therefore, to the extent that planned roadways are impermeable, reginal connectivity will drastically decline as linkages merely consolidate main patches and their nearby smaller satellites.

### 3.3 Opportunities for conservation and mitigation

**Fig 6:**
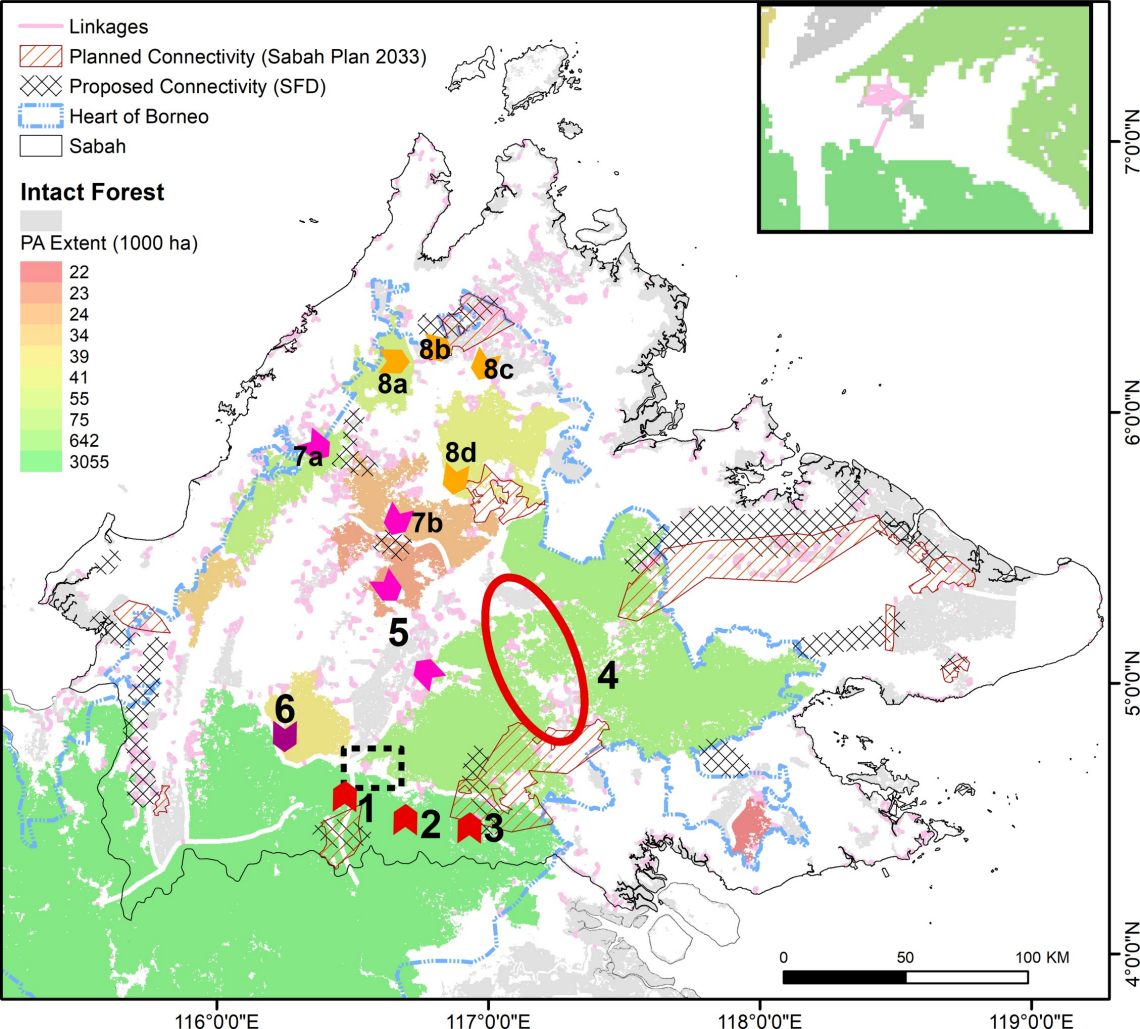
Select inter-patch links (numbered) by which forest corridors/underpasses would enhance the integrity of the northern Heart of Borneo region following planned road developments in Sabah. Note: Numbered arrows are indicative sites for corridor and road-underpass development that would enhance post-development forest/PA connectivity across Sabah and the northern HoB (S1 Table). The circled area at Site 4 is important for regional connectivity but would not require corridors/underpasses, just sound management alone (S1 Table). ‘Planned connectivity’ zones were adopted by the Sabah Structure Plan 2033 Proposal Map endorsed by Sabahan government in 2016 (plan SSP2033 Gazette LXXI/47/231) [36]. Planned connectivity zones near site 1 and in south-western Sabah approximate those of the Heart of Borneo Corridor Implementation Project [25, 54]. ‘Proposed connectivity’ zones are according to an earlier Sabah Forestry Department (SFD) proposal endorsed by the Sabah Structure Plan 2033 report [29]. PA extent is shown for the ten post-development intact forest patches with greatest PA extents. Intact forest patches are as defined previously. A corridor at Site 5 would bridge a series of disjointed patches lacking PAs (grey).

Following planned developments, any connectivity between the ‘confined’ trans-boundary patch in southern Sabah and the major patch to its immediate north-east (Fig 3B) would be by far the most important for regional integrity. The importance of linkages between these two main post-development patches is twice that of the next-most important linkage and nearly that of the next two most-important linkages combined, according to ΔIIC values. Such rankings underestimate the actual importance of connectivity between these two patches, considering that they would also host the greatest PA agglomerations accounting for one-quarter of HoB PA extent (Fig 4). The modelled connectivity between these two main patches is contingent on networks of stepping-stone patches along the Sapulut-Kalabakan route (Fig 6 sites 1, 2, 3). In practice, pro-active conservation planning would be essential to maintain these and other networks bridging critical main patches.

Fig 6 and S1 Table describe the indicative sites for corridor and underpass development that, from a structural perspective, would enhance post-development connectivity of intact forests and PAs across the northern HoB. The configuration of the indicative sites across Sabah is comparable to that of key post-development linkages where these linkages are permitted to span roadways (Fig 5B). The indicative sites also complement, but differ more markedly from, currently planned and recently proposed forest connectivity zones advanced by the Sabah Structure Plan 2033 (Fig 6). This is noteworthy considering that planned connectivity zones according to the Sabah Structure Plan 2033 aspire to more and larger forest corridors (>1.5 km wide), as well as more underpasses, compared to the past and to connectivity zones proposed by the Sabah Forestry Department, also endorsed by the Sabah Structure Plan 2033. Indeed, the Plan instructs that “an overpass or underpass shall be constructed to minimize ecological impacts” wherever a highway “cuts through forest connectivity”, particularly where it is a “national highway or strategic road” [29]. The 12 indicative sites differ from the planned and proposed connectivity zones of the Plan largely because the latter often do not account for impending losses of connectivity that would follow from planned infrastructure development (e.g., sites 5, 6, 7b, 8c in Fig 6).

Theoretically, corridors and underpasses at the indicative sites would retain ~85% of intact-forest connectivity after road development, as estimated by the proportion of the sum of all ΔIIC values for all linkages accounted for by the sites. Approximately half of this retained connectivity would be attributable to sites 1–3 and 5 alone, flagging these as priority areas (S1 Table), with only the former recognized by planned and proposed connectivity zones (Fig 6). The corresponding retention of PA connectivity would also be considerable: sites 8a-8d, 7b, and 5 would integrate 42% of PA extent in Sabah and, if complemented by sites 1–3, PA connectivity would theoretically extend from northern Sabah to the larger HoB (Fig 4, S3 Fig). However, in reality, the actual degree of meaningful *ecological* re-integration possible via underpasses and corridors would be far more moderate than the structural metrics reported here, as discussed below. The indicative sites are therefore best understood as mitigative options of last resort that complement planned and proposed connectivity zones.

## 4.0 Discussion and conclusion

### 4.1 Improved planning for the Heart of Borneo

The Pan-Borneo Highway and related roadways in Sabah would unilaterally cut off the head of the HoB by isolating hundreds of thousands of hectares of Sabahan PAs and other intact forest. The greatest challenge to regional HoB integrity is the planned Sapulut-Kalabakan upgrade-highway through the trans-boundary forest patch of southern Sabah (Fig 4).

Roadways for the Pan-Borneo Highway were largely planned independently of the planned and proposed connectivity zones in Sabah. Calls for a trilateral HoB Master Plan resonate in this light [23]. A HoB Master Plan would constitute the further formalization and codification of the original HoB trilateral agreement. Amongst transboundary conservation initiatives internationally, formalized transboundary joint governance is not uncommon ([20]:Appendix B). Related master plans for conservation and development are however arguably observed more amongst contiguous transboundary areas than expansive regional zones such as the HoB. The Marittime Alps-Mercantour transboundary conservation zone (France and Italy) and the Great Limpopo Transfrontier Park (Mozambique, South Africa, and Zimbabwe) both exemplify the evolution of informal joint management activities amongst national entities into formalized, common legal governance regimes entailing seamless transboundary plans ([20]:p.52,68). For the HoB, at a minimum a basic master plan could designate high-conservation forests, corridors, and forest buffer zones across the HoB with trilateral consensus. Such an overarching plan would in turn support related calls for the legal recognition of the HoB for planning purposes [55] and, further still, for trilateral spatial planning of conservation, land use, and infrastructure within the HoB [23]. Trilateral planning for common conservation-and-development goals across the HoB is considered to be much more effective and efficient than current approaches, according to conservation-scenario analyses [17, 18].

Compromised ecological planning across the HoB region partly reflects the fact that the HoB is a voluntary agreement lacking force of law. In Malaysia, infrastructure development schemes are designed federally (e.g., National Physical Plan) but elaborated by quasi-independent state plans (i.e., Sabah Structure Plan), within which the ‘HoB vision’ must be approximated under existing environmental laws and policies. The ultimate elaboration of state plans at the local level further challenges the regional HoB vision, as in instances where laws are conflicted or poorly observed. Similar situations characterize Indonesia and Brunei [55]. A trilateral HoB Master Plan would supplant such ‘good-will implementation’ and is feasible, depending on its ultimate scope and legal framework. A basic overarching trilateral Master Plan that identifies regional priority conservation networks and corridors, guides unilateral infrastructure and land-use plans accordingly, and more clearly articulates the responsibilities of state-level stakeholders may alone suffice to promote greater regional integrity. Intensive trilateral coordination, and possibly the legal recognition of HoB boundaries, may prove essential, since any master plan would invariably be the culmination of a common governance regime rather than a means to this end. More detailed trilateral spatial planning of infrastructure and land use across the HoB is far more challenging, and probably unlikely, given state sovereignty issues. Still, member states could conceivably call upon a basic overarching master plan to pre-emptively inform and hedge against unilateral development plans that would undermine regional HoB integrity as described by the plan. For this, a permanent HoB Secretariat, rather than the current annually-rotating trilateral Secretariat scheme, would likely be necessary to ensure a more consistent, rapid, and equitable coordination of the HoB conservation agenda.

### 4.2 Limited options for post-development mitigative conservation

Planned road development in Sabah relies on highway underpasses and forest corridors to retain connectivity across the HoB. Our analysis suggests that Sabah’s current plans overlook various sites where such ‘linear conservation’ would be beneficial. Regardless, the linear-conservation approach is dubious and risky, given limited evidence that species of high conservation value would benefit enough from this approach to offset the direct and indirect impacts of forest-road development [56, 57]. Such an approach is regarded as an ‘engineering solution’ to a conservation problem, as tokenistic, or more cynically as a strategic overture to conservation interests [56–58].

Peninsular Malaysia has previously employed forest corridors alongside roadway underpasses as local conservation measures, and Sabah is apparently increasingly adopting this approach. Peninsular Malaysia utilized 25 highway underpasses in conjunction with 17 forest corridors to integrate fragmented conservation landscapes via the Central Forest Spine initiative [35, 58, 59]. To date, Sabah has developed corridors to a lesser degree [60], at least with respect to large networks of corridors, and underpasses have rarely featured in these efforts. This situation may change with the implementation of the Sabah Structure Plan 2033, which explicitly advocates both greater corridor development and extensive roadway underpasses. This advocacy coincides with independent efforts to establish forest corridors across the HoB. The HoB Corridor Implementation Project, for instance, would establish corridors between at least a dozen PAs extending from south-central and southwestern Sabah into Sarawak, Kalimantan, and Brunei [25, 61]. Indeed, planned connectivity zones in southern Sabah according to the Sabah Structure Plan 2033 approximate those of the HoB Corridor Implementation Project.

Underpasses and corridors are unlikely to be effective mitigating conservation options in Sabah in light of the nature and scale of planned infrastructure development there. In the Central Forest Spine initiative in Peninsular Malaysia [35, 59], none of the highway underpass locations were based on surveys of animal movements or biodiversity [58]. Underpasses were instead simply a byproduct of normal road construction, whereby roads necessarily traverse rivers, creeks and gullies and so yield ‘underpasses’. Observers of Sabah anticipate a similar commitment to underpass development there, which would offer only limited benefits for faunal movements. Surveys of mammalian movements across 20 underpasses along two roadways of ~30–40 km length in Peninsular Malaysia suggest that underpasses facilitated meaningful road crossings for only two species (elephants, serows) of seven species observed, with only one of these species (serows) using the highway underpasses at expected rates ([59]:p.114-159). Mammals otherwise cross roads where and when they wish, increasing mortality [62], or they avoid roads, increasing population isolation [35]. Others have similarly concluded that linear conservation is at best an uncertain, if not ineffective, means of promoting meaningful ecological connectivity [56, 57].

Furthermore, constructing extensive networks of underpasses and related corridors explicitly for wildlife movement is likely prohibitively expensive, given the scale of planned developments. The costs of planning and constructing under- or over-passes in Singapore and Peninsular Malaysia have been estimated at $1.2-$32 million USD per 100 meters [63, 64]. Hypothetically, two 200-meter long underpasses at the eight indicative sites in Sabah likely requiring an underpass (S1 Table) would cost $38-$1024 million USD. Even the lower figure is greater than the Sabah HoB budget allocated by the Ministry of Natural Resources and Environment [22]–raising doubts over the feasibility of extensive linear conservation at regional scales. Although it is not inconceivable that underpasses could facilitate ecologically-meaningful exchanges amongst some forest patches, this would entail various carefully-planned underpasses per site, in conjunction with local faunal surveys, roadside fencing [62], and monitoring programs, further increasing costs and doubt over the feasibility of this approach. Additional costs for law enforcement to prevent roadside poaching would also be required, given poachers’ tendency to target specific species at underpass ‘bottlenecks’ [59, 65].

Other, complementary forms of mitigative conservation planning include enhanced conservation designations for roadside forests [66]. Limited scope remains for this in Sabah. Notwithstanding scattered agricultural holdings that are possibly permanent fixtures in any conservation landscape, all indicative sites for potential corridor and underpass development except site 7a are largely situated within forests designated for permanent protection (Fig 6, S1 Table), including Class II Commercial Forest Reserves. Class II reserves are however subject to partial conversion to timber plantations [67]–a trend that may gain momentum as roads are built and upgraded–and if these reserves are further degraded some may ultimately become re-designated for estate agriculture. Notably, the Sapulut-Kalabakan route separating the two main patches (Fig 4) is currently a poorly maintained, sealed former logging road to be upgraded to a four-lane highway surrounded by Class II reserves. Selectively precluding roadside timber plantations within such reserves, or elevating such reserves to Class I Protection Forest Reserves, remain viable options. This will not preclude impending losses of regional connectivity but merely hedge against its subsequent aggravation.

Recently, Sabah’s Chief Minister affirmed that planned roadway developments should occur along existing routes only, and not extend to intact forest areas, reflecting productive engagement between State agencies and an alliance of local conservationists [68]. While commendable, this fails to reconcile the fact that the planned upgrade of the existing Sapulut-Kalabakan route is by far the preeminent threat to the regional integrity of PAs and intact forests across the northern HoB. In other contexts, others have also highlighted how such major road upgrades typically accentuate deforestation by permitting new economic activities, all-season access to forests, and lower transportation costs for extracted resources [69]. Invariably, this dissonance between the recent decision by the Chief Minister and the continued planning of road upgrades reflects the multiple priorities and tradeoffs inherent to conservation and development planning. Conservationists, for their part, have focused more on sensitive, threatened local habitats and their resident endangered fauna, and less on regional forest integrity. Nonetheless, in light of the considerable loss of integrity posed by the Sapulut-Kalabakan route, we similarly urge the reconsideration of its development within the Pan-Borneo Highway network.

### 4.3 The Pan-Borneo Highway in the context of infrastructure mega-projects

The emphasis on infrastructure and conservation planning in the tropics has arguably shifted over recent decades from the preservation of intact forests to the integrated management of degraded landscapes. This shift reflects both the progressive ecological degradation within the tropics but also the increasing scale and complexity of infrastructure mega-projects and the contexts that host them. The Pan-Borneo Highway within the Heart of Borneo is a case in point.

Historically, the academic community highlighted the implications of major forest-penetration roads for intact-forest fragmentation [70–72] leading to subsequent habitat conversion and wildlife poaching [65]. In contrast, recent assessments of infrastructure development and conservation have emphasized the threats posed to remnant forests [7, 28], including protected areas [8, 9]. In settled landscapes, remnant forests often constitute the final frontiers for resource extraction or transportation impediments for expanding populations. Their vulnerability to infrastructure development has therefore been characterized increasingly as planning failures rather than as a carefully weighed ‘cost of development’. Even for regions with large expanses of intact forest, such as in New Guinea, recent assessments have focused more on the adequacy of environmental planning to contain conservation challenges arising from infrastructure development, and less on relatively foreseeable challenges posed by forest fragmentation along penetration roads [12, 73]. Studies of the ongoing Chinese Belt and Road Initiative (BRI) in particular stress the growing complexity of conservation challenges stemming from mega-projects in a globalized world in which environmental safeguards are frequently lax [10, 74].

Our assessment of the Pan-Borneo Highway resonates with recent BRI assessments [10, 11, 74, 75] in emphasizing environmental governance, and not merely environmental management, as central for sound mega-project development. Sabah offers at least two lessons in this respect. First, Sabah underscores the persistent need to more fully integrate conservation-and-development planning, legislatively and administratively. In the absence of such planning, conservation efforts will be undone and opportunities squandered by the sheer scale of development. Sabah, for instance, has more than doubled its coverage of protected forests since joining the Heart of Borneo, with concomitant increases in forest connectivity. Yet the Pan-Borneo Highway, promulgated independently of these conservation efforts, would limit or even reverse many of their conservation benefits. Second, integrated planning should occur at the most basic of scales to anticipate and forestall cumulative effects at regional scales. In Sabah, planned upgrade roads would disrupt regional conservation integrity as much as planned new roads (e.g., the Sapulut-Kalabakan route); yet upgrade roads are not equally subject to planning scrutiny. In fact, environmental impact assessments for upgrade roads are relaxed where ‘upgrades’ entail new highway construction parallel to an existing rudimentary road.

The amplification of planning deficiencies to national or multi-national scales by the BRI and similar initiatives has culminated most recently in unprecedented calls to unite conservation and development priorities into a single rubric [10, 11, 75]. The BRI and similar initiatives would, in effect, adopt conservation as a ‘core value’ or an explicit goal. The case of the Pan-Borneo Highway offers few clear examples of how this might look relative to older models seeking simply to protect intact forests from encroaching development. Sabah’s recent decision to avoid new road development in intact forests [68] resonates with calls for ‘no net biodiversity loss’ with respect to the BRI [11], although it does not drastically differ from the older models. The legal recognition of the Heart of Borneo and a trilateral Master Plan may guide development according to conservation priorities, although this would not necessarily support such priorities. Proposals for new networks of protected areas and corridors along infrastructure routes might afford greater synergies between conservation and development [11], though the anticipated isolation of PAs around the Pan-Borneo Highway urges caution. In the realm of road development at least, threats to regional connectivity in particular may remain a serious challenge without a clear planning patch.

## 5.0 Conclusion

Sabah is planning a Pan-Borneo Highway network to increase economic activity, as are variously other member states of the HoB initiatives. While Sabah has substantially increased its PA extent, the currently planned network would seriously undermine PA and intact-forest connectivity within Sabah and across the northern HoB region, reducing the conservation benefit of individual, isolated PAs and managed forest landscapes. Trilateral spatial planning across the HoB is recommended to forestall and hedge against such outcomes, but it will require coordination amongst HoB member states at a level not yet attained. Meanwhile, Sabah’s commitment to underpasses and forest corridors for conservation mitigation is arguably a futile gesture. It is apparently too geographically selective, too unlikely to facilitate meaningful ecological re-integration, and ultimately too economically costly as a practical solution. We urge that the Sapulut-Kalabakan route within the Pan-Borneo Highway network be reconsidered in particular.

## Supporting information

S1 Fig The extent of proposed new and upgrade roadways in Sabah according to the Sabah Structure Plan 2033, displayed relative to official forest protection status and designations, intact forest areas, and protected areas. Notes: ‘Forest Status and Designation’ is according to the Sabah Forestry Department. ‘Protected Areas’ are as defined in the main text, namely as the combined extents of the World Database on Protected Areas and the ‘protected’ forest-use designations observed by the Sabah Forestry Department.
(TIF)

S2 Fig **The integral index of connectivity (top) and corresponding number of networks of intact forest patches and linkages (bottom), for increasing greater inter-patch faunal dispersal distance thresholds across Sabah, northern Kalimantan, and eastern Sarawak. ‘Roaded’ and ‘unroaded’ curves respectively describe scenarios in which planned roadways are and are not developed.** Notes: The study area is delineated in Fig 5.
(TIFF)

S3 Fig Protected areas relative to potential sites for forest corridors/underpasses that would support the integrity of the northern Heart of Borneo region following planned Sabahan road developments. Notes: Numbered arrows are indicative sites for corridor and road-underpass development that would enhance post-development forest/PA connectivity across Sabah and the northern HoB (S1 Table). The circled area at Site 4 is important for regional connectivity but would not require corridors/underpasses, provided sound management alone (S1 Table). PA extent is shown for the ten post-development intact forest patches with greatest PA extents. A corridor at Site 5 would bridge a series of disjointed patches lacking PAs (grey).
(TIF)

S1 Table Conditions, costs, and contributions of complementary sites for road-underpass and forest-corridor planning in support of connectivity of intact forests and protected areas (PAs) across post-development Sabah. (DOCX)
