# Supplementary figures and images for: Trans-national conservation and infrastructure development in the Heart of Borneo
Source: PLoS One. 2019 Sep 18;14(9):e0221947. doi: 10.1371/journal.pone.0221947 (PMC6750574; doi:10.1371/journal.pone.0221947)

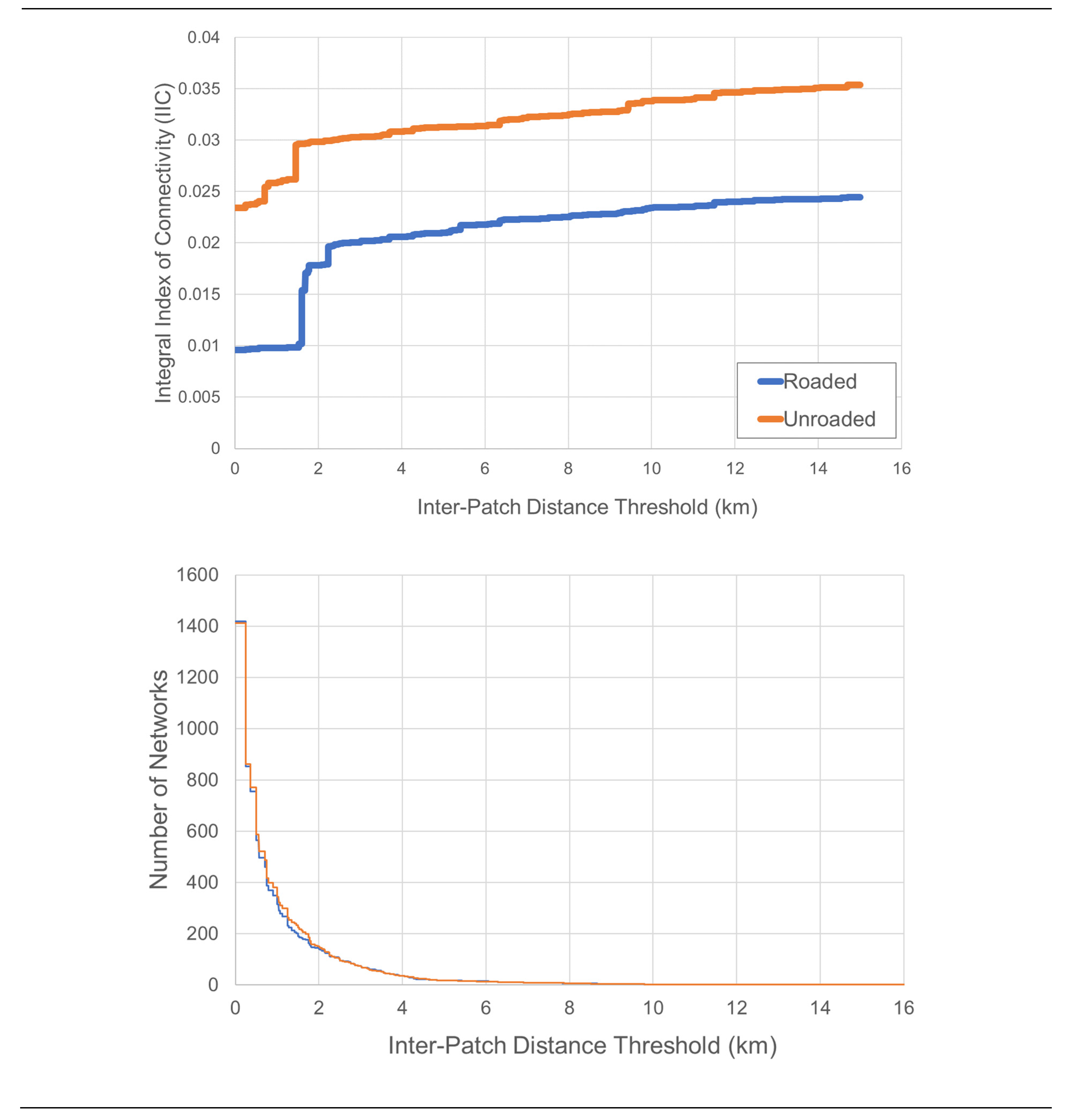

Supplement: S2 Fig — The integral index of connectivity (top) and corresponding number of networks of intact forest patches and linkages (bottom), for increasing greater inter-patch faunal dispersal distance thresholds across Sabah, northern Kalimantan, and eastern Sarawak. ‘Roaded’ and ‘unroaded’ curves respectively describe scenarios in which planned roadways are and are not developed. Notes: The study area is delineated in Fig 5. (TIFF) [file pone.0221947.s002.tiff]

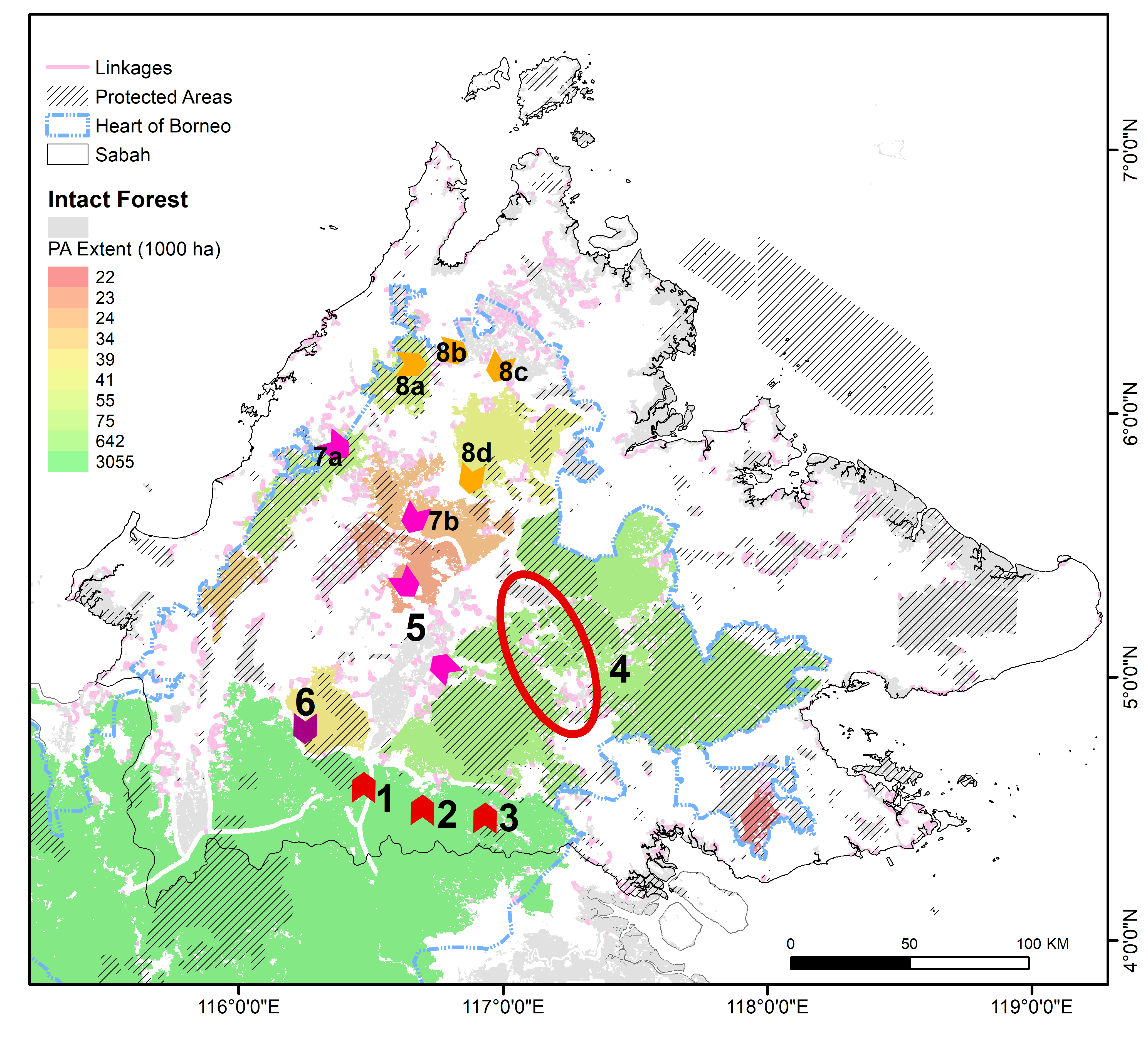

Supplement: S3 Fig — Notes: Numbered arrows are indicative sites for corridor and road-underpass development that would enhance post-development forest/PA connectivity across Sabah and the northern HoB (S1 Table). The circled area at Site 4 is important for regional connectivity but would not require corridors/underpasses, provided sound management alone (S1 Table). PA extent is shown for the ten post-development intact forest patches with greatest PA extents. A corridor at Site 5 would bridge a series of disjointed patches lacking PAs (grey). (TIF) [file pone.0221947.s003.tif]
