# Supplementary material for: Trans-national conservation and infrastructure development in the Heart of Borneo
Source: PLoS One. 2019 Sep 18;14(9):e0221947. doi: 10.1371/journal.pone.0221947 (PMC6750574; doi:10.1371/journal.pone.0221947)
Supplement: S1 Table — (DOCX) [file pone.0221947.s004.docx]

**S1 Table. Conditions, costs, and contributions of complementary sites for road-underpass and forest-corridor planning in support of connectivity of intact forests and protected areas (PAs) across post-development Sabah.**

| ***Site^a^*** | ***Current Forest Protection Status^b^*** | ***Conditions at Site to Traverse^b^*** | ***Potential Corridors and Underpasses*** | | ***Connectivity Importance (Greatest=100)^e^*** |
| --- | --- | --- | --- | --- | --- |
|  |  |  | *Costs and Logistics^c^* | *Contribution to PA and Intact Forest Connectivity^d^* |  |
| **1-3** | **High, Mixed.** Class II Commercial Forest Reserve at south of roads. Class I Protection Forest Reserve and buffer zones at north and south of site 3. | Existing unsealed low-traffic road, pending upgrade to 4-lane highway; heavily forested. | **Low to Moderate.** Underpasses; possible corridor restoration amongst stepping-stone patches, depending on roadside development intensity. | **Extremely High.** Would support bulk of PA connectivity between Sabah and larger HoB. | 100 |
| **4** | **Moderate.** Class II Commercial Forest Reserve spans the area. | Heavily logged with minimal human incursion. | **Very Low.** Proper management and recovery of logging reserve. | **High.** Would support integrity of northern main post-development patch and corresponding contributions to the larger HoB. | n/a |
| **5** | **Moderate, Mixed**. Class I Protection Forest Reserve at east, and Class II Commercial Forest Reserve at west. | Planned new highway, where no major public roads exist; heavily logged extensive forest with peripheral oil-palm plantations. | **Low to High.** Underpass(es) coincident with planned roadwork; costs increased if decommissioning plantations to ensure ample corridor where narrow (<3km). | **Moderate to High.** Would extend bulk of HoB connectivity into central Sabah, connecting an additional 23 Kha PA. | 53 |
| **6** | **High.** Class I Protection Forest Reserve spans the site. | Planned new highway, where no/few public roads exist (depending on route); heavily forested, accidented landscape. | **Low to Moderate.** Underpass(es) coincident with planned roadwork. | **Moderate to High.** Would extend bulk of HoB connectivity, connecting an additional 39 Kha PA. | 13 |
| **7a** | **Nil.** ~12-km wide area not officially designated as forest for management, between Crocker Range National Park (west) and Class I Protection Forest Reserve (east). | Existing sealed, high-traffic roadway(s) with dense roadside settlement, agriculture; patchy ~2 km-wide forest corridor at northeast of Crocker Range National Park. | **High to Very High.** Multiple underpasses for existing roads, possibly bridging northern parts of Crocker Range Park; agricultural decommissioning, land-use rezoning, corridor restoration, road reconstruction. | **High.** At minimum would connect 75 Kha of isolated western highland PA with 24 Kha PA (central Sabah), and potentially to larger HoB region, pending activities at site 5. | 5 |
| **7b** | **High, Mixed.** Small Class I Protection Forest Reserve at site, with Class II Commercial Forest Reserves at north and south. | Planned new highway, where no/few public roads exist (depending on final route); heavily forested, accidented landscape. | **Low to Moderate.** Underpass(es) coincident with planned roadwork. | **Moderate to High.** Benefits of activities at site 7b alone are modest, connecting only 24 Kha of PA to 23 Kha of PA immediately to the south. In conjunction with activities at sites 5 (first priority) and 7a (second priority), benefits are very high, connecting over 700 Kha PA thus across Sabah. | 5 |
| **8a** | **High, Mixed.** Class I Protection Forest Reserve (west) and Class II Commercial Forest Reserve (east) span the site. | Ongoing upgrade of narrow sealed road bordering Kinabalu Park; heavily forested, sparsely settled for ~4 km length. | **Low to Moderate.** Underpass(es), depending on traffic intensity and control measures (e.g., restricted traffic during evenings). | **Moderate to Extremely High.** Benefits from 8a alone are moderate, connecting 54 Kha of isolated western highland PA to a commercial forest reserve (north-central Sabah). Benefits are high in conjunction with 8b and 8c, connecting an additional 41 Kha of PA (central Sabah). Benefits are extremely high in further conjunction with sites 8d, 7b and 5, connecting Kinabalu Park to the larger HoB region. | 13 |
| **8b** | **Moderate, Mixed**. Class II Commercial Forest Reserve at northern fringe, with forest cover not designated for forest management along southern fringe. | No/few public roads; lightly logged, heavily forested mountainous Class II Commercial Forest Reserve, minimal human incursion. | **Very Low.** Proper management and recovery of logged reserve; monitoring peripheral agricultural activities and rudimentary unsealed track. | **Dependent.** See 8a above. | 5 |
| **8c** | **Moderate, Mixed**. Small Class 1 Protection Forest Reserves separated by oil palm, flanked by Class II Commercial Forest Reserves; 4-8 km gaps between reserves, notwithstanding limited interstitial forest cover. | Rudimentary unsealed road pending upgrade to highway; logged, mostly forested Class II Commercial Forest Reserve; extensive swidden fallows plus encroaching plantations. | **Low to Moderate.** Underpass(es) coincident with planned roadwork; rezoning swidden-agricultural area for corridor. | **Dependent.** See 8a above. Maximum benefit may entail additional connectivity enhancements spanning river parallel to road for small or aboral species, e.g., rope bridges for orangutans. | 5 |
| **8d** | **High, Mixed.** As with site 8c, but with smaller gaps amongst forest reserves (1-4 km) and greater interstitial forest cover. | Existing sealed mountainous road pending upgrade to 4-lane highway through Bukit Taviu Protection Forest Reserve; heavily forested along ~7 km length of roadway; extensive swidden fallows plus encroaching plantations. | **Low to Moderate.** Underpass(es) coincident with planned roadwork. | **Moderate to Extremely High.**  Benefits are moderate for 8d alone, connecting 41 Kha with 24 Kha of PA. Benefits are high in conjunction with 8a-8c and extremely high in further conjunction with 7b and 5 (see 8a above). | 62 |

Notes: (a) Colors correspond to site locations in Figs 6 and S3. (b) Protection Status: Class I Protection Forest Reserves are for environmental protection and biodiversity conservation. They are protected against all exploitation and conversion. Class II Commercial Forest Reserves allow logging but not conversion, excepting limited conversion to industrial timber plantations. (c) Costs and Logistics: Nominal appraisals reflect whether underpasses are necessary to span planned roadways, the local extent of settlement, planned road type and proximity to forest, current land-use designation (e.g., forest/conservation, agriculture), and extent and type of agriculture (e.g., oil palm plantation, small-scale farming). (d) Contributions to PA and Forest Connectivity: Nominal appraisals reflect the extent of PAs and intact forest area to a lesser degree that would be structurally connected via corridors and underpasses. (e) Connectivity Importance: Reflects ∆IIC values for inter-patch linkages from the patch in question to a nearby large patch as indicated in Figs 6 and S3. The ∆IIC values are rescaled to 1-100 due to their non-denominational nature, where 100 represents the greatest observed value. For sites 5, 8a, 8b, and 8c, reported values are sums for multiple linkages whereby multiple small, proximate patches serve as ‘stepping stones’ to link two main patches of interest. Such small patches are generally semi-contiguous or contiguous in reality and indicate heterogeneity in forest condition more than forest cover per se. Site 4 is degraded but requires no inter-patch linkages.
